# Case Report: Atypical Presentation of Large Bowel Obstruction Due to Bezoar Formation in a Patient with a History of Colon Cancer

**DOI:** 10.5070/M5.53997

**Published:** 2026-10-31

**Authors:** Amanda Li Nyam, Luke WallsSmith

**Affiliations:** * University of California, Irvine, Department of Emergency Medicine, Orange, CA; ^ Sutter Roseville Medical Center, Department of Emergency Medicine, Roseville, CA

## Abstract

Abdominal pain is one of the most common presenting complaints in emergency medicine. This case report summarizes a 61-year-old male with worsening abdominal pain and no bowel movement for multiple days. Computer tomography (CT) scan demonstrated a large intraluminal mass at the anastomosis, concerning for a bezoar formation, with associated stercoral colitis and partial bowel obstruction. The patient was subsequently admitted to the general surgery service for further management. During hospitalization, the patient’s stool burden improved with osmotic laxatives, and he underwent a colonoscopy. The patient was discharged with outpatient follow-up. This report will help inform management in a patient with a history of colon cancer presenting with a large bowel obstruction post-colectomy.

**Topics:**

Colon cancer, bezoar formation, stercoral colitis, bowel obstruction.

**Figure:**
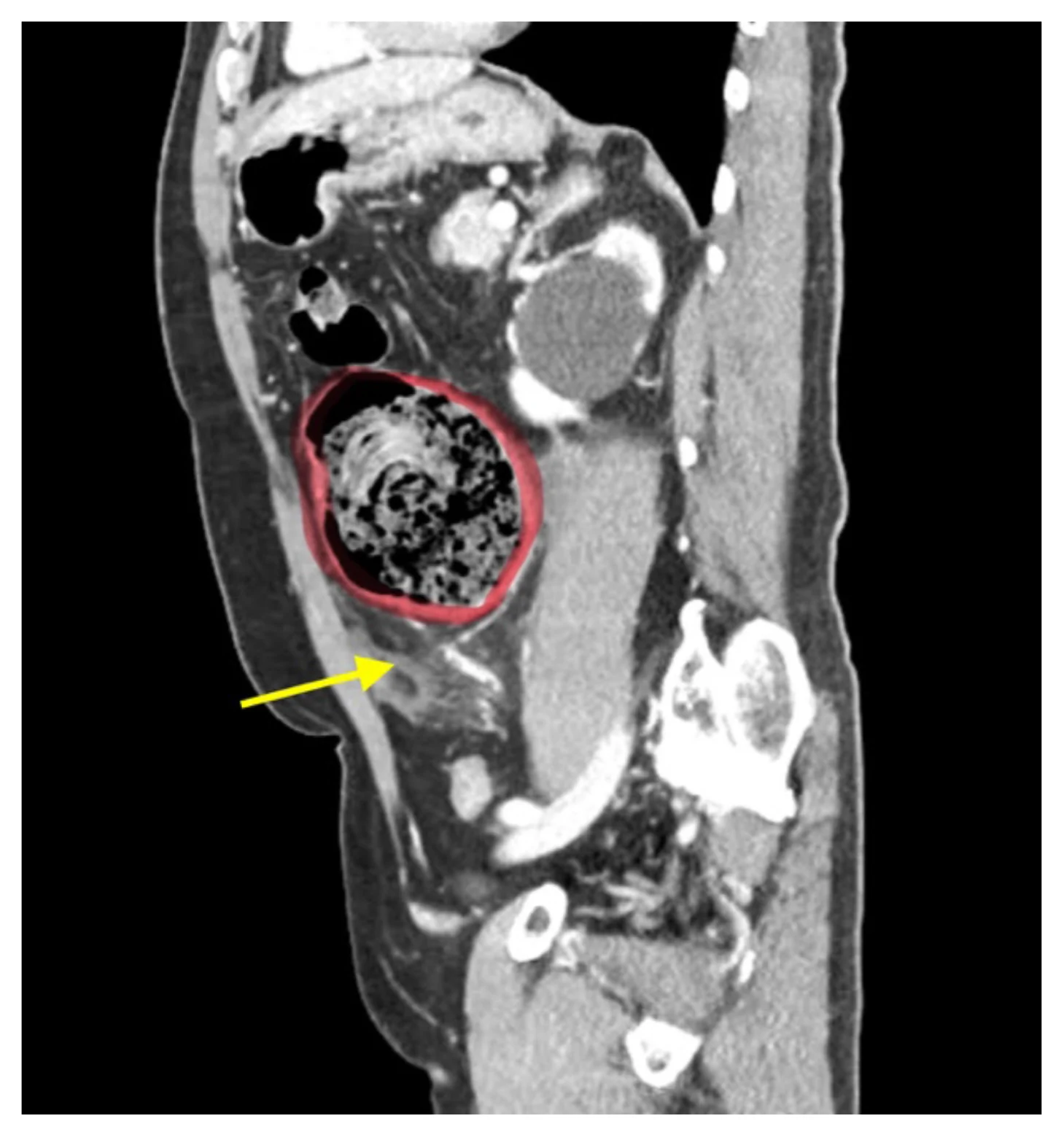


**Figure:**
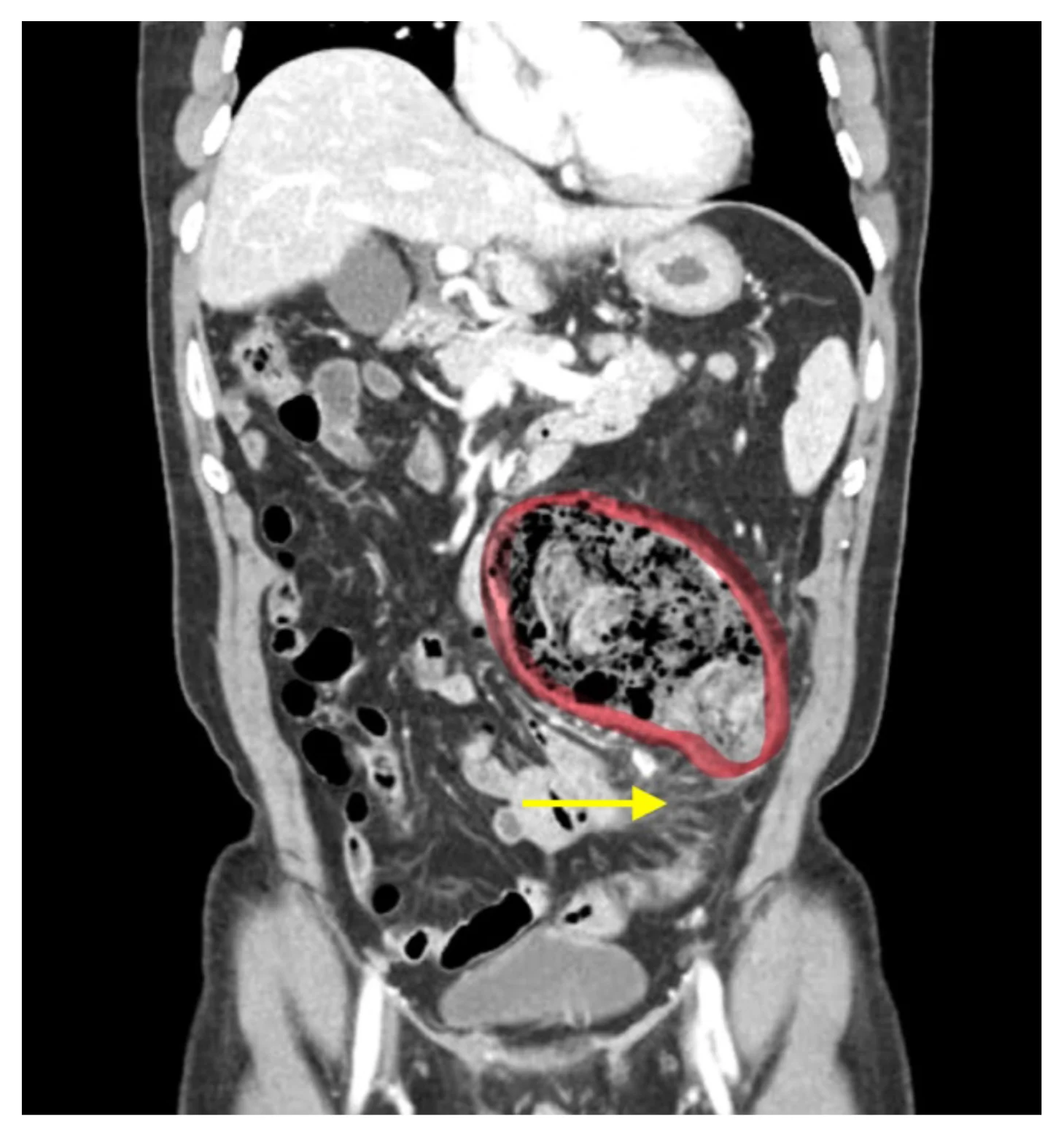


**Figure:**
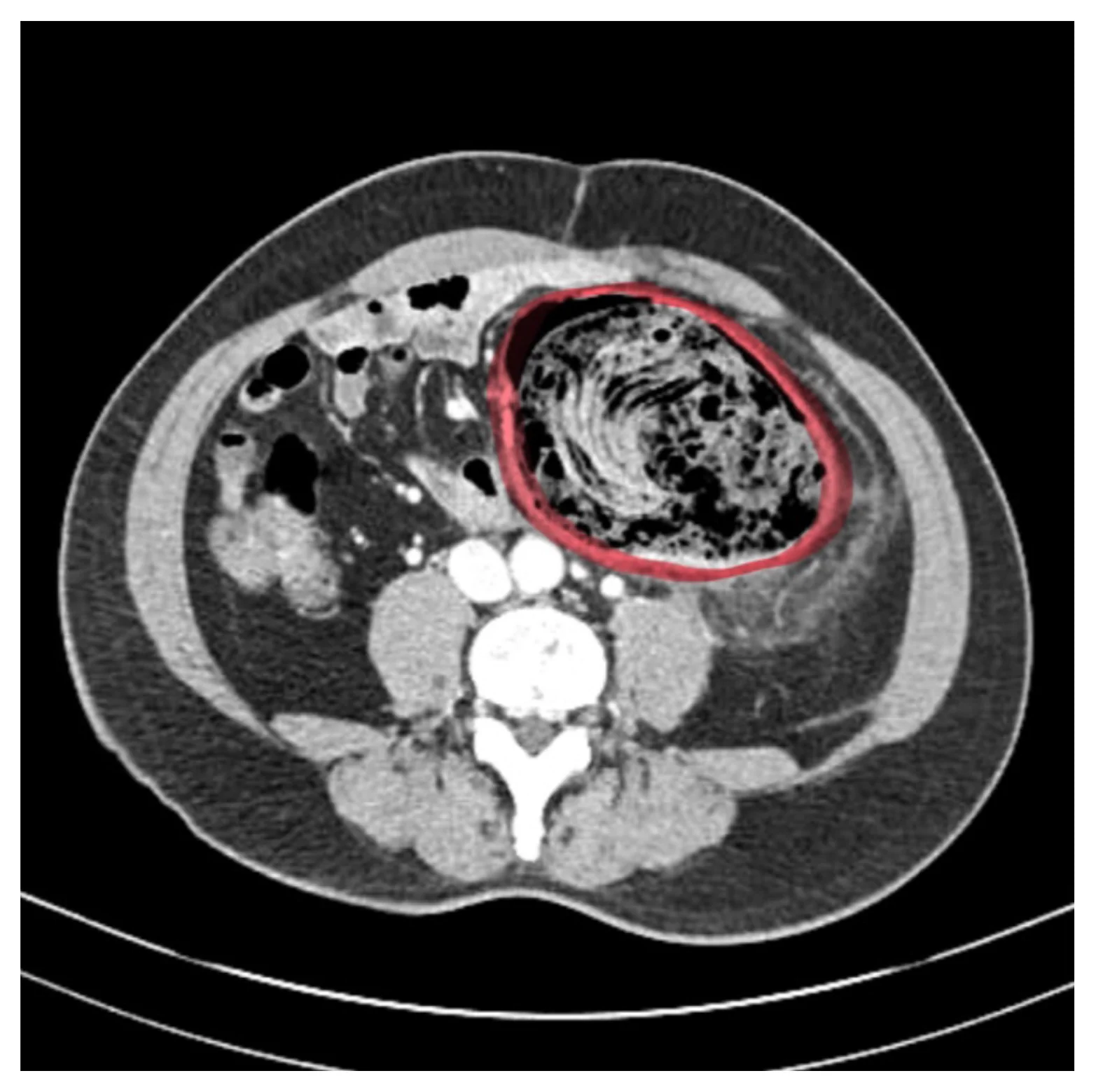


**Figure:**
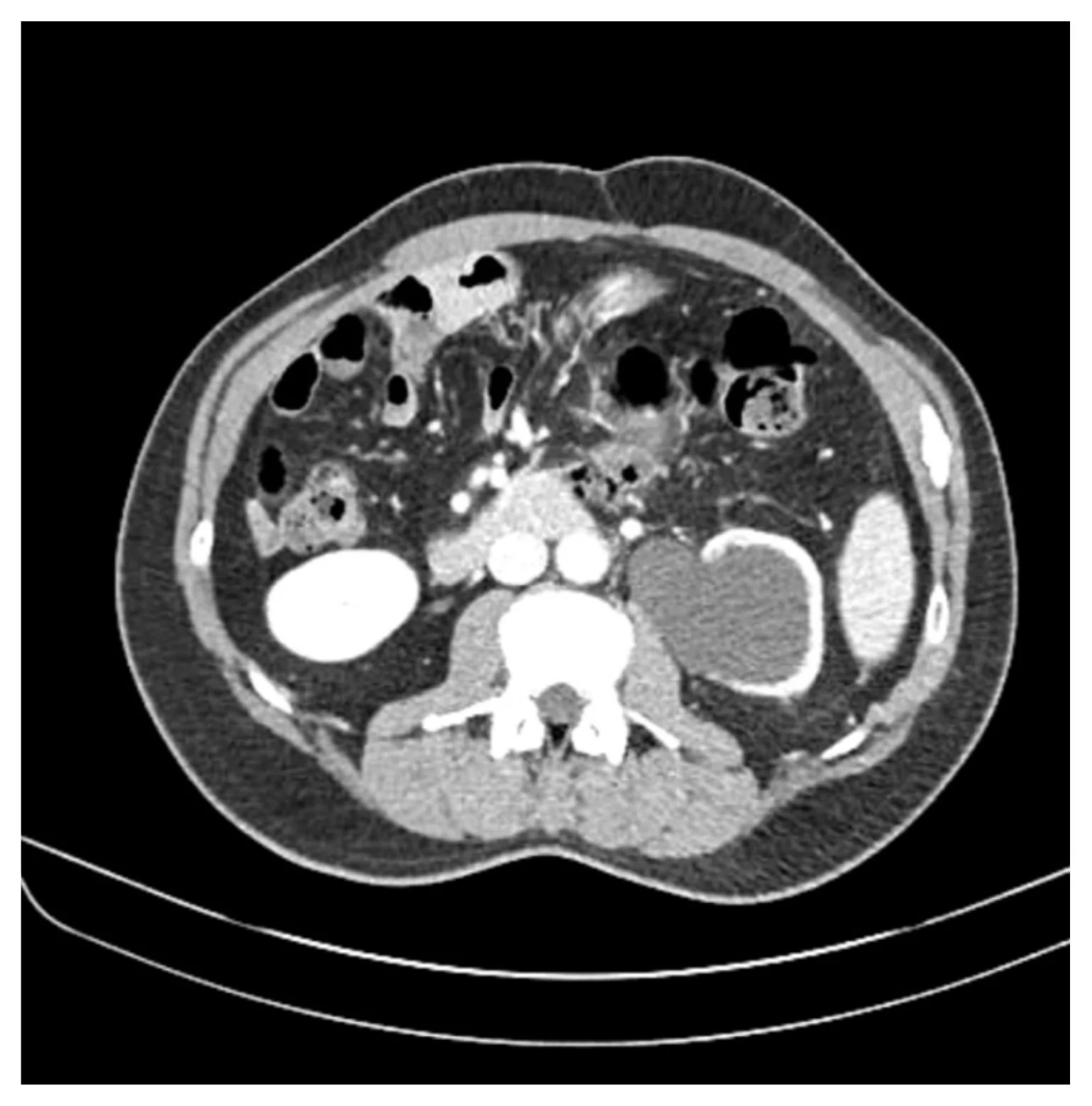


## Brief introduction

Acute abdominal pain accounts for 5–10% of all emergency department (ED) visits.^1^ The differential diagnosis is broad. Therefore, a thorough history and physical exam can help delineate the cause of symptoms along with appropriate labs and imaging. Bowel obstructions are a frequent cause of hospitalization and surgical intervention, and large bowel obstructions (LBOs) account for 20–25% of obstructions.^2^ Benign anastomotic stricture is a recognized complication after colorectal surgery, with reported incidence ranging from 5 to 30%; however, the incidence of LBOs attributable to these anastomotic strictures is less well defined.^3^,^4^ In our patient, we observed findings suggestive of stercoral colitis secondary to an LBO, specifically a bezoar, a concretion of indigestible material in the gastrointestinal tract. It is essential for emergency medicine physicians to recognize LBO with associated stercoral colitis as a cause of abdominal pain in a patient with a history of colon cancer and partial colectomy. Stercoral colitis is a relatively uncommon but serious inflammatory condition caused by pressure from fecal impaction or other intraluminal material.^5^ It occurs most often in the rectosigmoid region in older adults and in those with chronic constipation, immobility, neurologic disease, or opioid use.^6^ It may also develop in patients with post-surgical colonic anatomy or mechanical obstruction. If left untreated, stercoral colitis can progress and lead to ulceration and perforation.^6^ In this patient, stercoral colitis developed in association with an LBO. Failure to recognize an LBO or bezoar formation-induced LBO with associated stercoral colitis can delay treatment and increase mortality.^7^

## Presenting concerns and clinical findings

The patient was a 61-year-old male with a past medical history of colon cancer in the splenic flexure now status-post partial colectomy, complicated by a small bowel obstruction with lysis of adhesions and exploratory laparotomy who presented with generalized abdominal pain along with lack of bowel movements for three days. He was able to pass flatus without issue. Vital signs were within normal limits. On exam, there was a palpable mass in the left lower quadrant of his abdomen with associated tenderness.

## Significant findings

Lab results were notable for marginally elevated creatinine from baseline and no leukocytosis. CT with intravenous (IV) contrast revealed: (1) a large amount of fecal material in the descending colon at the level of anastomosis suggesting bezoar formation, (2) colonic wall thickening and inflammation suggestive of stercoral colitis, (3) abrupt caliber change at distal descending colon indicating partial large bowel obstruction, and (4) moderate to severe left hydronephrosis due to ureteropelvic junction obstruction with 0.4 cm calculus. No abscess or pneumatosis visualized. Annotated images demonstrate a large intraluminal mass at the descending colon anastomosis with associated abrupt caliber change (arrow), causing partial LBO and adjacent colonic wall inflammation (highlighted) consistent with stercoral colitis.

## Patient course

General surgery was initially consulted given the CT findings and recommended admission for further evaluation and management of obstruction. On further interview, the patient revealed that he did not have a follow-up colonoscopy after his partial colectomy and diagnosis of cancer. His last visit with his oncologist was 2 months prior and his carcinoembryonic antigen (CEA) test at that time was slightly elevated, suggesting that the patient’s obstruction and bezoar may be due to recurrence of cancer. Polyethylene glycol was started, and gastroenterology was consulted for a possible colonoscopy, which was performed during hospitalization. Samples taken during the colonoscopy did not reveal evidence of infection or malignancy. The patient was discharged with outpatient follow-up.

## Discussion

This patient’s course highlights the diagnosis and management challenges of a partial LBO in a patient with a prior colectomy for colon cancer. The abrupt caliber changes and wall thickening at the anastomosis on CT raised suspicion for both benign and malignant etiologies. Although the obstructing lesion was initially described as a bezoar on CT, a large fecaloma or fecal impaction could not be excluded; the distinction between the two is not always straightforward. Bezoars are compactions of indigestible material such as plant fiber or medications that accumulate and solidify within the gastrointestinal tract, whereas fecal impaction refers to retained, dehydrated stool.^8^,^9^ In the colon, these can overlap radiographically. The term “bezoar” was applied in this case based on the spongy-appearing intraluminal mass with organized peripheral air pockets rather than the diffusely mottled gas pattern typical of fecal impaction, in which small air pockets are scattered throughout the stool and conform to and fill the colonic lumen.

Stercoral colitis, as seen in this patient, further represents complications of altered colonic anatomy post-colectomy. In this patient, the bezoar at the anastomotic site served as the cause of stercoral colitis; it can precipitate or worsen obstruction and may lead to colonic ulceration and necrosis, perforation, or ischemia if left untreated.^6^,^7^,^10^,^11^ Bezoar-induced stercoral colitis is rare and should not be excluded, especially in post-surgical patients with altered gastrointestinal motility.^12^ In mild cases, stercoral colitis can be managed with a digital rectal disimpaction and bowel enema or oral bowel regimen.^3^,^13^,^14^ Opioids should be avoided due to constipation as a side effect; antiemetics and fluids may also be given. If perforation, ischemia, or sepsis is suspected, surgery should be consulted and IV antibiotics covering gastrointestinal flora should be initiated.^3^,^5^,^13^,^15^

The sensitivity of CT for detecting colorectal cancer as the cause of a large bowl obstruction is 91–95% and specificity is 91–99% in the context of mechanical obstruction with most false positives due to benign strictures or mass-like lesions such as diverticulosis or fecal impaction.^16^,^17^ Recurrent malignancy and anastomotic strictures are both recognized to be causes of post-colectomy bowel obstructions; it is important to differentiate between both to guide therapy.^18^,^19^

Colonoscopy can be both diagnostic and therapeutic. There have been instances of successful colonoscopic decompression and catheter drainage for postoperative obstruction at the anastomosis site.^19^ In this case, a colonoscopy was warranted to evaluate for recurrent malignancy or anastomotic stricture, and immediate relief could be offered if a benign cause such as constipation was confirmed.^20^

In summary, the management of a partial LBO with associated stercoral colitis in a post-colectomy patient requires a multidisciplinary approach, with imaging and colonoscopy serving as tools for diagnosis and intervention. Early identification of the underlying cause is key to guiding therapy and improving patient outcomes.

## Supplementary Information










